# Self-Assembled Gold Nano-Ripple Formation by Gas Cluster Ion Beam Bombardment

**DOI:** 10.3390/ma10091056

**Published:** 2017-09-08

**Authors:** Buddhi P. Tilakaratne, Quark Y. Chen, Wei-Kan Chu

**Affiliations:** 1 Department of Physics and Texas Center for Superconductivity, University of Houston, 4800 Calhoun Rd, Houston, TX 77204, USA; qchen@faculty.nsysu.edu.tw (Q.Y.C.); wkchu@uh.edu (W.-K.C.); 2 Department of Physics and Center for Nanoscience and Nanotechnology, National Sun Yat-Sen University, Kaohsiung 80424, Taiwan

**Keywords:** ion-solid interaction, cluster ion beam, nano-ripples

## Abstract

In this study, we used a 30 keV argon cluster ion beam bombardment to investigate the dynamic processes during nano-ripple formation on gold surfaces. Atomic force microscope analysis shows that the gold surface has maximum roughness at an incident angle of 60° from the surface normal; moreover, at this angle, and for an applied fluence of 3 × 10^16^ clusters/cm^2^, the aspect ratio of the nano-ripple pattern is in the range of ~50%. Rutherford backscattering spectrometry analysis reveals a formation of a surface gradient due to prolonged gas cluster ion bombardment, although the surface roughness remains consistent throughout the bombarded surface area. As a result, significant mass redistribution is triggered by gas cluster ion beam bombardment at room temperature. Where mass redistribution is responsible for nano-ripple formation, the surface erosion process refines the formed nano-ripple structures.

## 1. Introduction

The fabrication of metal nano-scale structures on surfaces has generated great interest in surface, optical, and biomedical engineering applications: for example, surface wetting, nano-tribology, surface plasmon resonance-based applications, such as surface-enhanced Raman scattering, catalytic surfaces, and bio-sensors, and optical waveguides. The fabrication of nanostructures with homogeneous spread with controlled dimensions is always a challenge. Chemical techniques such as sol-gel [1] and electrochemical deposition methods [2] are extensively used to fabricate metal nanostructures, but the presence of chemical contaminants is a concern even with selective chemical solutions. Techniques, such as laser ablation deposition, sputtering deposition, and thermal evaporation, reduce the contamination; however, nanostructures are non-uniform without proper lithographic or physical masking [3,4,5,6,7].

Another approach is to use ion beams directly to induce nanostructures by means of self-assembly. Nano-ripple structures formed by ion beam bombardment-induced self-assembly on material surfaces was first investigated by Navez et al. in 1962 using a single ion beam [8]. However, a lack of high resolution microscopic characterization techniques slowed the nano-scale surface studies until recently. In 1988, Bradley and Harper proposed a theoretical model (BH model) [9] for surface pattern formation with the association of Sigmund’s theory of sputtering [10] According to their model, the ripple formation occurs due to local-area surface curvature-dependent erosion and the thermal diffusion of target atoms. Their model was modified over the past two decades to adopt crystallographic, and material dependencies of surface patterns created by single ions [11,12,13,14,15,16]. In addition, Carter and Vishnyakov, in their article published in 1996, discussed major contributions for surface pattern formation as mass redistribution due to momentum transfer from single ion to target atoms [17]. In 2009, Norris et al. considered contributions to the surface pattern formation as a two-part process, a prompt regime, accounting for single-ion collision, and a gradual regime, which assumes the relaxation of structures due to the viscous flow of atoms. They took into account contributions from the prompt regime, which is designated by surface erosion and mass redistribution, considering a crater function that assumes many impacts at a location, collectively inducing a small local change in height from a flat surface due to the formation of a crater, to develop a continuum equation to describe the nano-pattern formation. In this model, they assumed, compared to the nano-structure height, that the local height change is extremely small [18]. Experimental investigations conducted by Madi et al. used the contribution of surface erosion and mass redistribution to describe the nano-pattern evolution on silicon substrate surfaces [19].

Here, we utilized gas cluster ion beam (GCIB) to produce nanostructures on substrate surfaces. A GCIB consists of thousands of atoms, which are bound by van der Waal’s forces. The GCIB-induced nanostructure formation possesses a broad-beam mechanism that is different compared to single ion bombardment. Since the energy per atom in the gas cluster ion is only a few electron-volts, the cluster ion–target material interactions are limited to near the surface compared to single ions, which is ideal for surface smoothing processes that can reduce the surface roughness down to the sub-nano-scale, when the GCIB incident normal to the target surface. The gas cluster ions provide high throughput, and the irradiation process is shorter relative to a single ion beam. Therefore, since the inception of GCIB experimental studies by Yamada and co-workers in early the 1990s [20], research and development has significantly increased as an industrial tool for surface smoothing along with numerous other applications [21]. Interestingly, in contrast to surface smoothing process, off-normal incidence of GCIB bombardment induces surface roughness. Continuous off-normal angle bombardment of a target surface form nano-scale ripples that closely resemble macroscopic Aeolian sand-ripple patterns [22]. A recent study conducted by Lazano et al. showed that GCIB off-normal bombardment on Si (100), (110), and (111) form surface nano-ripples independent of crystal orientation of the Si substrate, and that nano-ripples are oriented perpendicular to the surface vector of the incident gas cluster ion with a fixed azimuthal angle [23]. Further, Toyoda et al. showed that nano-ripple self-assembly is independent of the cluster ion size when energy remains constant per cluster ion for fluences lower than 1 × 10^16^ clusters/cm^2^ [24].

In this article, we investigate the formation and the correlation of surface erosion and mass redistribution to the GCIB bombardment-induced self-assembly of nanostructures on gold surfaces with different incident angles and applied fluences under room temperature conditions. We characterized GCIB-bombarded surfaces by measuring the nano-ripple dimensions by atomic force microscopy (AFM), measuring the surface sputtering yield by Rutherford backscattering spectrometry (RBS), and finally analyzing features on and between nano-ripples by scanning electron microscopy (SEM).

## 2. Experimental Procedure

**Figure 1:**
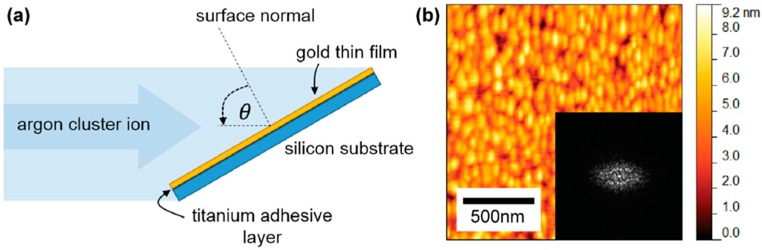
(**a**) The schematic diagram of the experimental setup and (**b**) non-bombarded polycrystalline gold surface.

Gold substrates used for this study were polycrystalline thin films deposited on Si surfaces (Sigma Aldrich, Inc., St. Louis, MO, USA). A Ti adhesive layer is used to bind a gold film on to the Si (100). Gold surfaces were bombarded using an Epion gas cluster ion beam accelerator with 30 keV Ar cluster ions averaging 3000 Ar atoms per cluster. Therefore, each atom in a cluster carries about 10 eV of energy. The mechanism of generating Ar-GCIB by supersonic expansion of Ar gas and a detailed description of the production of gas cluster ions is explained elsewhere [25]. Figure 1a shows the schematic diagram of primary configuration of the experimental setup. Initially, samples were bombarded at varying incident angles from 0 to 80°, measured from the surface normal, in steps of 10° to determine the angular dependence of self-assembly process of gold nanostructures, where fluence was kept at a constant value of 1 × 10^16^ clusters/cm^2^. During the experiment, the beam current density was kept at 1 μA/cm^2^ to minimize beam heating. To investigate the fluence dependence, the applied fluence was varied from 1 × 10^15^ to 3 × 10^16^ clusters/cm^2^ for three selected incident angles of 50°, 60°, and 70°. 

In each case, gold surface morphologies were characterized with an AFM system (Park Scientific Instruments, Santa Clara, CA, USA) in contact mode covering an area of 1.6 µm × 1.6 µm. The AFM tip used in this study was NANOSENSORS™ PPP-CONTR-10 with a radius of curvature less than 10 nm, and the silicon cantilever was coated with aluminum on the detector side. The AFM data were analyzed using Gwyddion open source software. The surface root mean square roughness ($R_{rms}$) was extracted from AFM micrographs where $R_{rms}$ is calculated as the statistical change of height ($h_{k,l}$) at a point on the surface, $R_{rms}=\;{\left\{\left(\frac{1}{L}\right)\overset{N}{\underset{l=1}{\sum }}\overset{M}{\underset{k=1}{\sum }}{\left[h_{k,l}-<h>\right]}^{2}\right\}}^{1/2}$ [26], where <h> is the average height of points on the surface. 2D fast Fourier transform (FFT) images of AFM micrographs were obtained to determine the variation of the dominating structural pattern, here a windowing function, known as Hann function was used to suppress the data at the edge of image, w(β)=0.5−0.5cos(2πβ), where β is an independent variable within the range (0,1) [27]. A 2D FFT image consists a centered zero frequency point, a point corresponding to the structures exhibited in an AFM image, and an identical out-of-phase point. For a perfect sine wave pattern, there should be one point on either side of the centered zero frequency point, whereas for an irregular pattern there will be a distribution of frequencies on either side of the zero frequency point. The thickness of the radial distribution of these frequencies describes the order of nanostructures shown in the AFM micrographs. The dominant wavelength (λ) perpendicular to the nano-ripple pattern is extracted from the 2D FFT of each AFM micrograph. For a non-bombarded gold surface as shown in Figure 1b, $R_{rms}=1.28\;\mathrm{nm}$ and λ=85 nm.

The effective macroscopic sputtering yield was determined by RBS using a 1.7 MV tandem accelerator (National Electrostatic Corporation, Middleton, WI, USA, 5SDH) with a 2.0 MeV alpha particle beam. The beam spot size was 1.0 mm and sputtering yield was estimated by calculating the energy difference (∆E) between alpha particles backscattered from the surface of gold film and the titanium–gold interface, which is related to the remaining gold thin film thickness. The thickness (t), $t=\;∆E/N\varepsilon_{o}$, where $\varepsilon_{o}=2.21\;\times \;10^{-16}\;\mathrm{keV}/\left(\mathrm{atoms}/{\mathrm{cm}}^{2}\right)$ is the stopping cross-section factor, and $N=5.90\;\times \;10^{22}\;\mathrm{atoms}/{\mathrm{cm}}^{3}$ is the density of the gold thin film. Inherent surface morphological features were further evaluated using a scanning electron microscope system (FEI, Hillsboro, OR, USA).

## 3. Results

**Figure 2:**
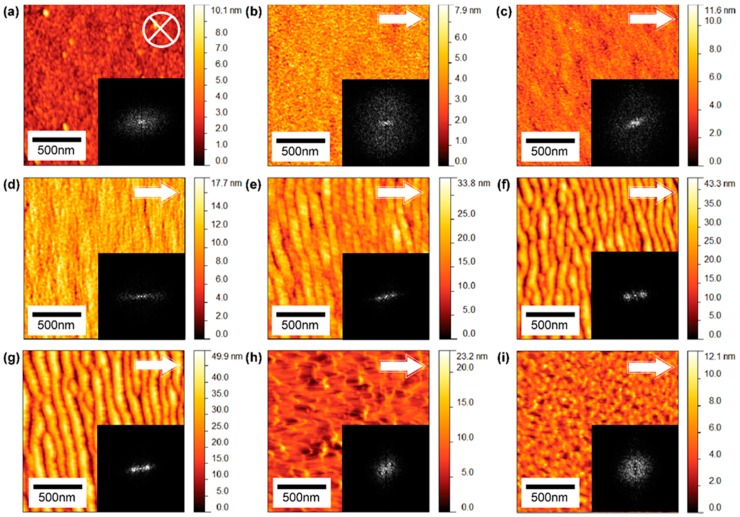
Atomic force micrographs of Ar-GCIB modified gold surfaces at different incident angles (*θ*) with fluence of 1 × 10^16^ clusters/cm^2^ and energy of 30 keV per cluster ion: (**a**) 0°; (**b**) 10°; (**c**) 20°; (**d**) 30°; (**e**) 40°; (**f**) 50°; (**g**) 60°; (**h**) 70°; (**i**) 80° from the surface normal. Inserted FFT images show the ordering and disordering of surface structures. The arrow denotes the direction of incident cluster ions. The scan area for all images is 1.6 × 1.6 μm^2^.

**Figure 3:**
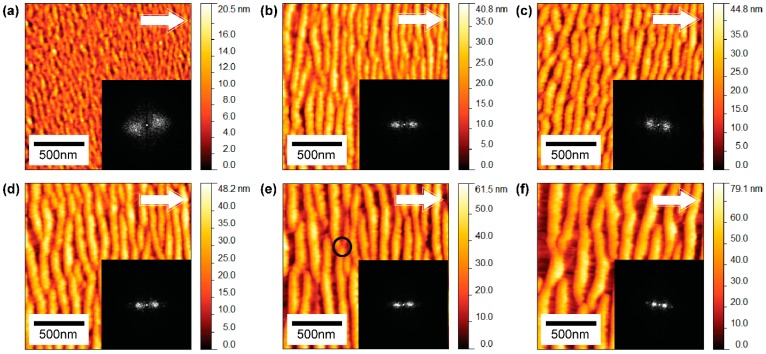
Atomic force micrographs of gold surfaces irradiated with Ar-GCIB at different fluences with 60° incident angle and energy of 30 keV per cluster ion (**a**) 0.1 × 10^16^; (**b**) 0.4 × 10^16^; (**c**) 0.6 × 10^16^; (**d**) 0.8 × 10^16^; (**e**) 2.0 × 10^16^; (**f**) 3.0 × 10^16^ clusters/cm^2^. The inserted FFT images show thinning of the radial width of the Fourier peak, which indicates the ordering of ripple structures with applied cluster ion fluence. The circle marked in (e) indicates a dislocation. The arrows denote the direction of cluster ions. The scan area for all images is 1.6 × 1.6 μm^2^.

AFM micrographs shown in Figure 2 represent morphologies of gold surfaces bombarded with GCIB at different incident angles recorded from 0 to 80° for an applied fluence of 1 × 10^16^ clusters/cm^2^. Nano-ripples are prominent between 40 (Figure 2e) and 60° (Figure 2g). Below 40°, AFM micrographs nanostructures are not observed, while above 60° nanostructures become irregular due to momentum transfer to the target gold surface decreasing, inducing only kinetic roughening (Figure 2h). Figure 2 insets show 2D FFT images. The thickness of the radial width of the centered Fourier peak describes the order of the surface nanostructures generated during GCIB bombardment; for a perfect periodic nanostructure, the radial distribution width is small and has sharper points on either side of the centered point. Nano-ripples formed at 60° are well-ordered and show similar frequency points on either side of the centered zero frequency point. Figure 3 shows the AFM micrographs of surfaces bombarded with increasing fluence of GCIB at an incident angle of 60°. Figure 3 insets show corresponding 2D FFT images that illustrate the ordering of nanostructures. During this ripening process, nano-ripples grow with applied fluence by superposition of smaller nanostructures and dislocations (as shown in Figure 3e, the black circle denotes dislocations of nano-ripples). 

**Figure 4:**
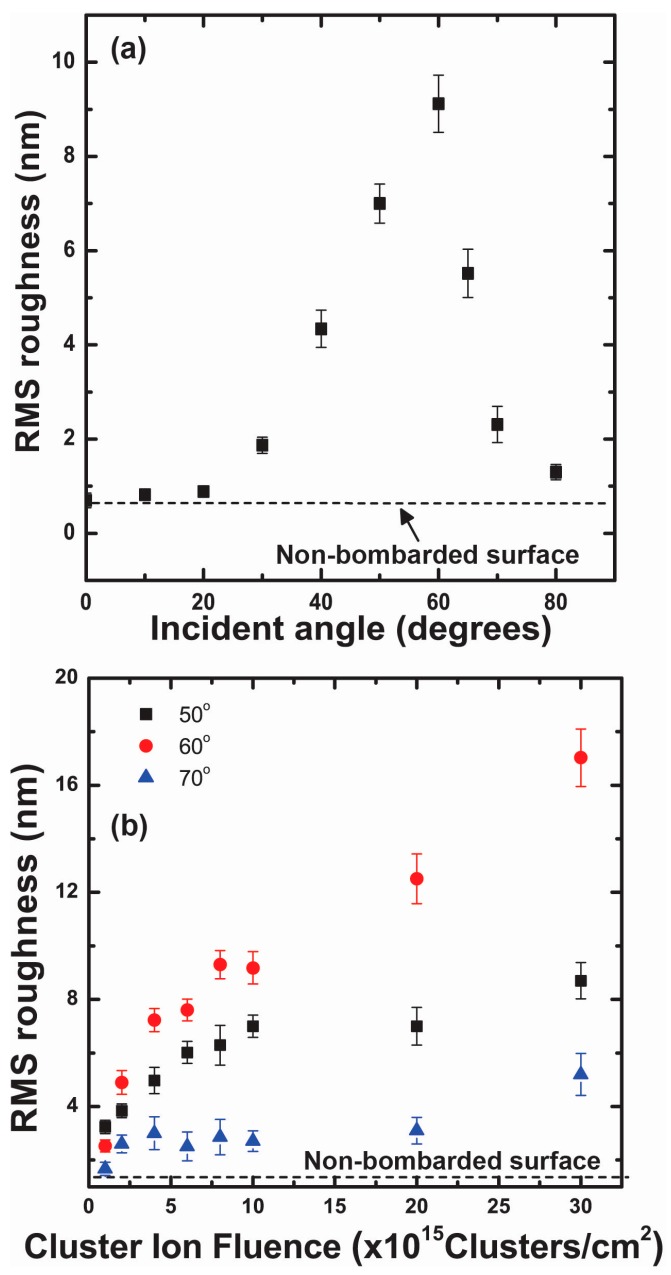
(**a**) RMS roughness of the Au surface with the cluster ion incident angle. After this critical angle of 60°, the surface roughness reduces considerably; (**b**) RMS roughness at the critical angle of 60° remains higher than that at 50° and 70° with fluence.

**Figure 5:**
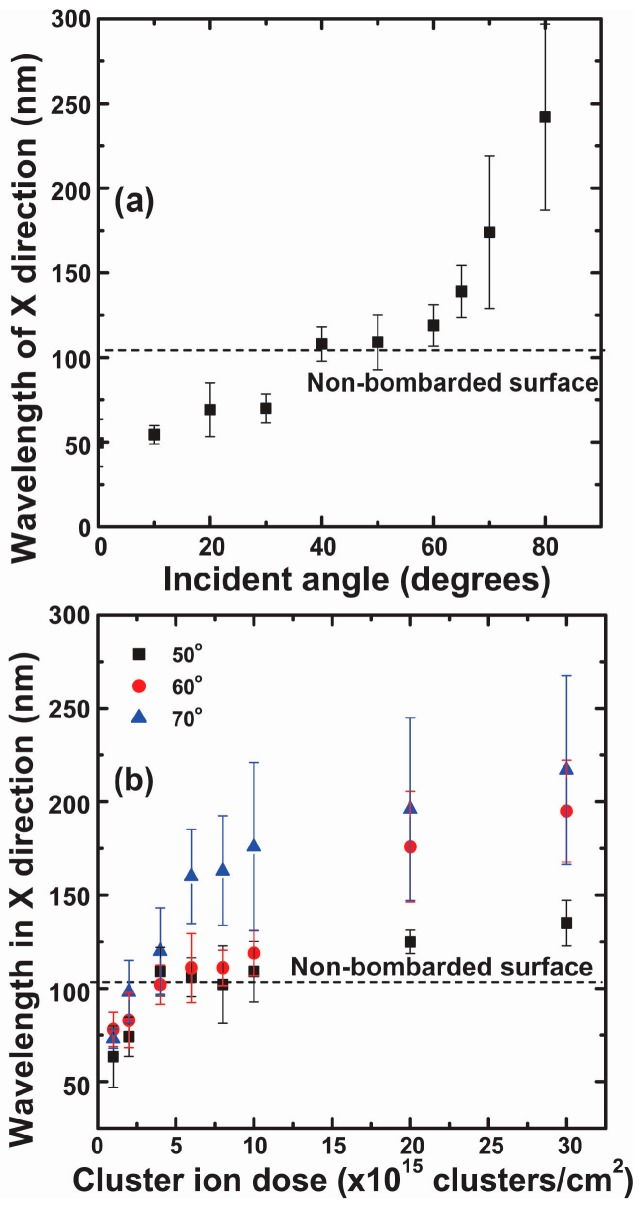
(**a**) The surface-dominant wavelength is measured with respect to the gas cluster ion beam incident angles measured from the surface normal. The wavelength exponentially increases with gas cluster ion beam incident angle; (**b**) Variation of the wavelength of nanostructures depending on the applied fluence of gas cluster ion beam for incident angles 50°, 60°, and 70°.

**Figure 6:**
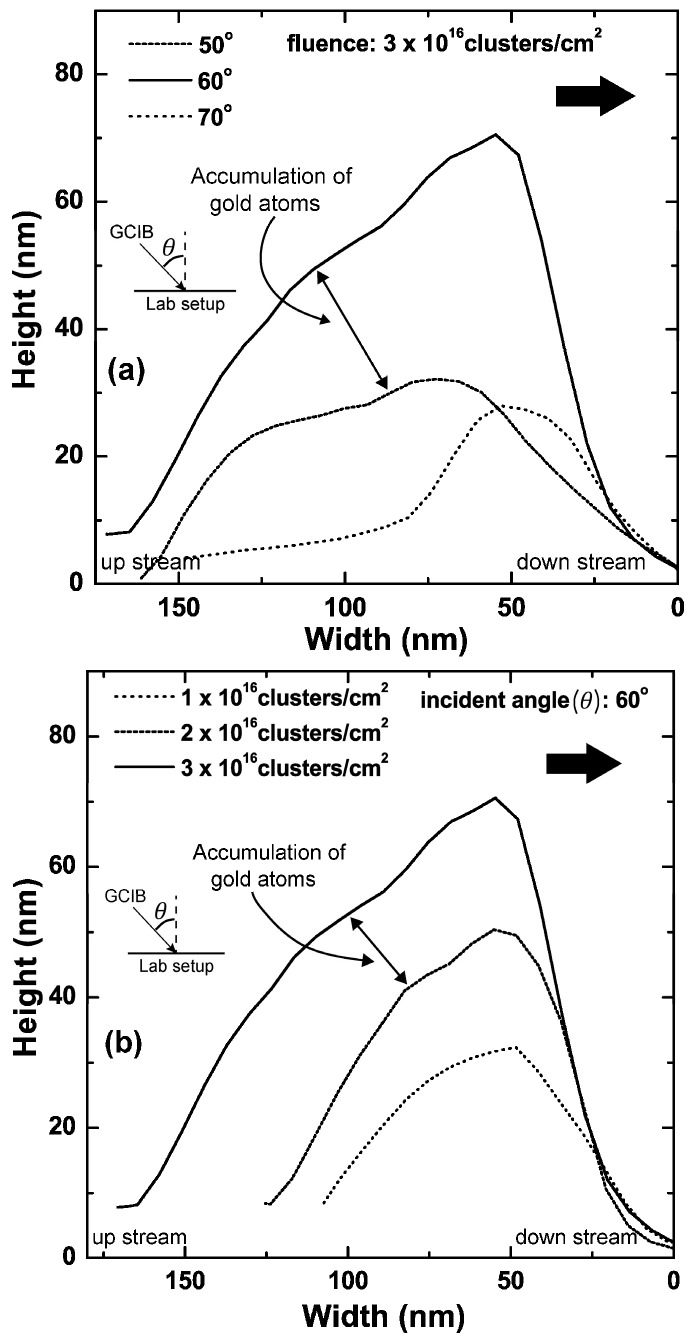
Comparison of cross sections of nano-ripples obtained from atomic force microscope images depending on (**a**) the GCIB incident angle and (**b**) the applied fluence of GCIB. The increase in the upstream of the nano-ripple is due to the atomic accumulation of surface atoms driven by mass redistribution and localized erosion.

Figure 4a shows the $R_{rms}$ of gold surfaces that were bombarded at different incident angles of GCIB ranging from 0 to 80°. Measurements indicate that, at 60°, $R_{rms}$ of the nano-ripple pattern is at its maximum. For a fluence of 1 × 10^16^ clusters/cm^2^, $R_{rms}$ was 9.12 nm and $R_{rms}$ increases to 17.03 nm when fluence increased to 3 × 10^16^ clusters/cm^2^. The $R_{rms}$ sharply increases between 20° and 60° and decreases beyond this critical incident angle of 60°. Figure 4b shows the fluence dependence of $R_{rms}$ for incident angles 50°, 60°, and 70°. The rate of change of $R_{rms}$ at 60° is greater compared to 50° and 70°. When the incident angle of GCIB changes from the surface normal to grazing incidence, the dominant wavelength of the surface nanostructures increases exponentially with the GCIB incident angle, as shown in Figure 5a. The dominant wavelength of the gold surface structures increases from 54 to 242 nm when the incident angle is varied from 10 to 80° for an applied fluence of 1 × 10^16^ clusters/cm^2^. The dominant wavelength also increases with applied fluence, but the rate of change in wavelength is higher for 70° compared to that for 50° and 60°, as shown in Figure 5b. Figure 6a shows the cross sections of nano-ripples formed during GCIB bombardment at an applied fluence of 3 × 10^16^ clusters/cm^2^ for incident angles 50°, 60°, and 70°. The black arrow indicates the direction surface component of the bombarding gas cluster ion. Figure 6b shows the growth of nano-ripple dimensions with applied fluence at the incident angle of 60°. The aspect ratio for the applied fluence of 3 × 10^16^ clusters/cm^2^ is around 50%.

**Figure 7:**
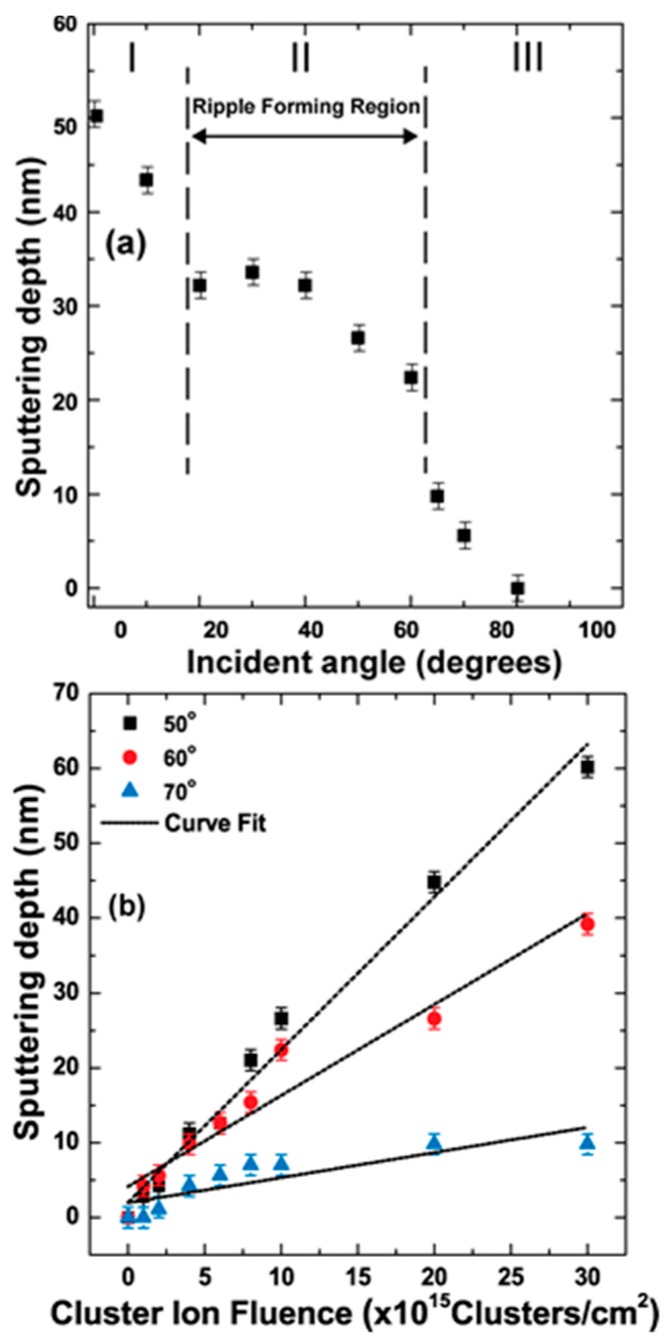
(**a**) Angular dependence of sputtering yield (depth) of Au thin film by Ar-GCIB at 1 × 10^16^ clusters/cm^2^ fluence. The amount of Au atoms removed is calculated with comparison to a non-bombarded Au thin film by RBS analysis. The sputtering yield variation with the cluster ion angle is classified into three regions; (**b**) Fluence dependence of sputtering yield for GCIB incident angles *θ* = 50°, 60°, and 70°.

The erosion rate of the gold surface during GCIB irradiation was characterized by sputtering yield, which is the average number of gold atoms leaving the surface per cluster ion impact. We measure the thickness of the gold film before and after bombardment of GCIB by RBS to calculate the sputtering yield. Figure 7a shows the average amount of gold film eroded with respect to GCIB angle of incidence at a fluence of 1 × 10^16^ clusters/cm^2^. The sputtering yield here decreases linearly with the angle of incidence from 0 to 20° (Region I) and from 20 to 60° (Region II), and the general sputtering yield resembles a cosine behavior. This shows that the population of gold atoms that attain the energy to escape the surface potential decreases within the Region II compared to Region I. The Region III (60 to 80°) erosion rate decreases due to cluster ions impacting at grazing angles that minimize the momentum transfer from cluster ion to the surface gold atoms. However, grazing angle incidence complicates the situation due to the fact that slowly moving cluster ions are attracted to their images of an opposite sign generated in metal and are to some degree deflected toward the surface. Therefore, the actual impact angle may be smaller than expected. To acquire the significance of the erosion rate due to the amount of cluster ions incident on to the gold surface, we measured the sputtering yield with respect to fluence for incident angles of 60°, 50°, and 70°, as shown in Figure 7b, which show that the sputtering yield of gold atoms follow a linear curve with applied fluence. The average erosion rates for GCIB incident angles 50°, 60°, and 70° are 12, 7, and 2 gold atoms per cluster ion impact.

**Figure 8:**
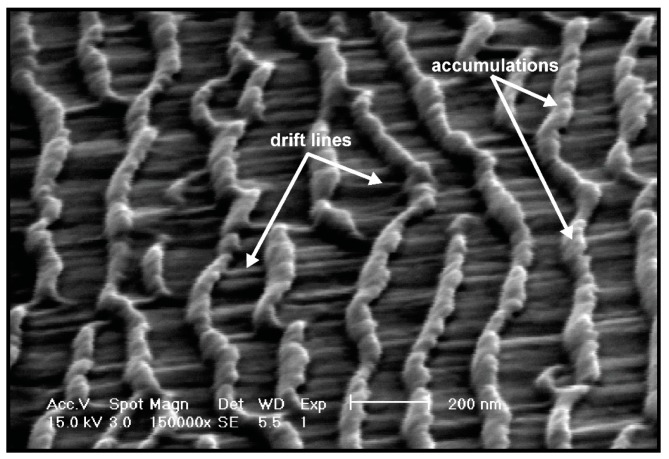
Scanning electron microscopy image of gold nano-rippled surface obtained by rotating the sample 70° from the electron beam gun. The ripple surface was prepared by gas cluster ion beam bombardment at an incident angle of 60° with a fluence of 3 × 10^16^ clusters/cm^2^.

We employed SEM imaging to further investigate nano-scale features on gold surfaces. The SEM sample stage was rotated such that the SEM electron gun was 70° from the normal of the gold surface. Figure 8 shows the SEM image of a GCIB-induced gold nano-ripple structure formed during bombardment with a fluence of 3 × 10^16^ clusters/cm^2^ at an incident angle of 60°. Between two nano-ripples, we observed drift lines parallel to the surface momentum component of the incident GCIB that were formed behind each nano-ripple structure.

**Figure 9:**
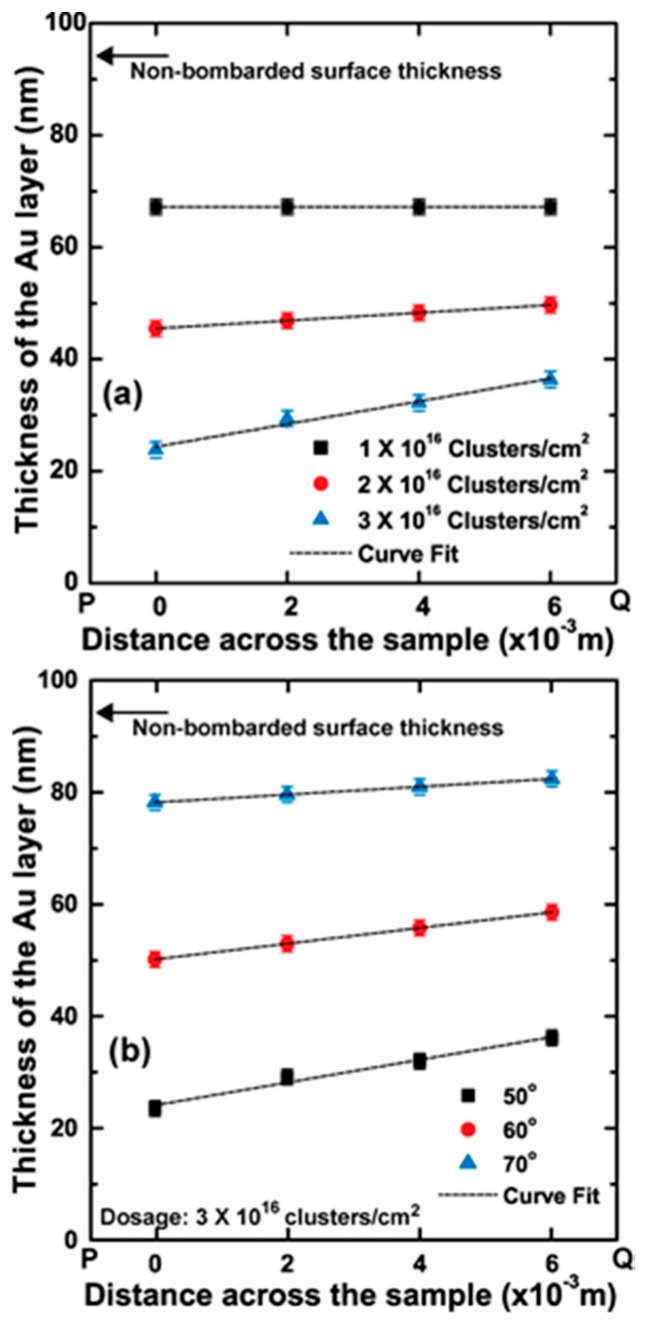
(**a**) The thickness of the Au thin film after bombardment with 1 × 10^16^, 2 × 10^16^, and 3 × 10^16^ clusters/cm^2^ at a 50° cluster ion angle of incidence. Thickness was measured 2 mm apart in the direction P to Q (cluster ions arrive onto the surface in this direction); (**b**) The thickness of Au thin film after bombardment with 3 × 10^16^ clusters/cm^2^ fluence at 50°, 60°, and 70° cluster ion beam incident angles. Thickness was measured 2 mm apart in the direction P to Q (the surface component of the cluster ion is in the PQ direction).

During RBS characterization of gold thin films, we determined that there were variations in thickness of the film of a sample when measured end to end in the direction perpendicular to the nano-ripple pattern, which was only visible for fluences greater than 1 × 16 clusters/cm^2^ due to the limiting resolution factor of RBS measurements. Figure 9a illustrates the gold film thickness across the surface at four points located 2 mm apart for fluences of 1 × 16 clusters/cm^2^, 2 × 16 clusters/cm^2^, and 3 × 16 clusters/cm^2^ at a 50° angle of incidence, and the gradient of these surfaces were 0, 7.0 × 10^−7^, and 20.3 × 10^−7^, respectively. When the angle of incidence of the GCIB increases from the surface normal, the gradient decreases as shown in Figure 9b. The figure illustrates the gradient formed due to GCIB bombardment at incident angles 50°, 60°, and 70° for a fluence of 3 × 16 clusters/cm^2^. The observed gradients for 60° and 70° compared to 50° were 14.0 × 10^−7^ and 7.0 × 10^−7^, respectively.

## 4. Discussion

A large gas cluster ion carries very low kinetic energy per atom, unlike a monomer ion; when it interacts with a target material, it generates thermal spikes in the target that experience high pressure and high-temperature transitions [28,29]. Molecular dynamic simulation studies conducted on the impact of large gas cluster ions with solid target surfaces show that the outermost atomic layers of a cluster ion create significant surface modifications [25,30,31,32,33]. However, a gas cluster ion transfers momentum to the target atoms when cluster impact generates shock waves within the cluster and in the target, and the dynamics of the shock controls the angular and mass energy transfer between the cluster ion and the target [34]. The magnitude of modification to the surface depends on the energy of the impact [35]. For a normal incident GCIB, surface smoothing of a target material occurs due to the lateral displacement of target atoms. 

Off-normal gas cluster ions transfer energy to the target atoms in the forward direction, here “forward” signifies the surface impact direction of the cluster ion beam in the laboratory frame. The key development of nano-ripples occurs between GCIB incident angles of 40° and 60°. Below 40°, nano-ripples are not observable due to the high surface erosion and less of a contribution to the coalescing process to form nano-ripples. When the GCIB incident angle approaches grazing incidence, it effectively removes surface mounts and smooths the surface without generating significant surface erosion; this smoothing effect is in agreement with previous studies on grazing incidence GCIB bombardment on surfaces [24,36,37].

In this investigation, we used as-deposited gold thin films as our starting substrates with 1.28 nm surface roughness; however, we determined that similar nano-ripple formations can be achieved from extremely smooth flat gold surfaces. Further investigations will be needed to determine what value of surface roughness would become relevant to create significant effects on the nano-ripple formation. The basic component that contributes to pattern formation due to GCIB bombardment is the formation of craters near the surface. Molecular dynamics simulation studies show that a fraction of target atoms accumulates on the front edge of the crater produced by the impact of the cluster ion at an off-normal angle, while for normal incident impact produces accumulations around the ring of a crater [25,28,31,32,33]. Continuous bombardment of gas cluster ions at a fixed off-normal angle leads to nano-ripple patterns oriented perpendicular to the general direction of the cluster ion beam impact. The amplitude of these atomic accumulations is extremely small compared to the nano-ripples, however, these accumulations trigger the basic direction of the nano-pattern. During the growth process, these accumulations superposition to form nano-ripples. The amplitude and separation of these structures are influenced by the applied fluence of GCIB, as illustrated in Figure 4 and Figure 5. Nano-structures show rapid growth with fluence initially, but slow down after a fluence of 5 × 10^15^ clusters/cm^2^ for GCIB incident angles 50°, 60°, and 70°. However, beyond this critical fluence, nano-ripples grow due to accumulation of gold atoms on the upstream of nano-ripples, as verified in Figure 6b. Gold atom accumulation decreases on the upstream of nano-structures for 70°, compared to that for 60° and 50°, as a result of GCIB shadowing due to the nano-structures formed at 70°. Even though we observe the accumulation on the upstream at 50°, high surface erosion affects the growth of nanostructures at this angle of incidence.

Behind each nano-ripple structure produced by cluster ion bombardment, we observed drift lines that developed during the gold atom movement on the surface, which is triggered by mass redistribution process along the general direction of the cluster ion impact (Figure 8). Drift lines suggest that target gold atoms migrate in the general direction of the cluster ion impact. The gradient buildup is a consequence of the directional gold atom migration resulted from the impact of the cluster ions; however, overall roughness remains consistent throughout the surface. The current induced by the impact of cluster ion beam may result in electro-migration that influence the bulk movement of target atoms [38]. Surface atoms that accumulate on the front edge of the ring of a crater are continuously pushed forward as a result of the continuous cluster ion bombardment. During this mass redistribution process, some of the surface atoms are continuously eroded because gold atoms have the potential to escape the surface. Thus, the nano-ripple ripening process is a coupling effect of the mass redistribution and the surface erosion. 

## 5. Conclusions 

We have given a description of off-normal GCIB bombardment-induced nano-ripple evolution on gold surfaces. The nano-ripple formation process is significantly governed by the GCIB incident angle and the applied fluence. The mass redistribution of surface gold atoms is affected by the bulk energy transfer due to the size of the gas cluster ion and atomic energy transfer due to the outermost atoms of the gas cluster ions. The growth of nano-ripples is caused by the atomic energy transfer, and these structures are pushed forward by the bulk effect. The sputtering yield has a cosine behavior with the GCIB incident angle between 40 and 60° as a result of the increased accumulation of atoms on the upstream of a nano-ripple structure. The localized erosion due to increasing surface curvature with applied fluence refines the nano-ripples. However, at grazing incident angles, the cluster ion to target atom energy transfer decreases, which reduces surface erosion and mass redistribution of target atoms. The crater function theory can be utilized to explain GCIB-induced nano-ripple formation with additional consideration of atomic and size effect of GCIB, which will provide the bridge between the microscopic and macroscopic structure evolution. Another future investigation that would be interesting is the determination of the effect of surface temperature on nano-ripple formation. Gas cluster ion beam bombardment is a useful technique to produce self-assembled periodic nano-ripples on gold surfaces, which provides a cost-effective single-step process with excellent repeatability.
